# Ambient Air Pollution and Age-Related Eye Disease: A Systematic Review and Meta-Analysis

**DOI:** 10.1167/iovs.63.9.17

**Published:** 2022-08-12

**Authors:** Alyssa Grant, Gareth Leung, Ellen E. Freeman

**Affiliations:** 1 School of Epidemiology and Public Health, University of Ottawa, Ottawa, Ontario, Canada; 2 Ottawa Hospital Research Institute, Ottawa, Ontario, Canada

**Keywords:** air pollution, age-related eye disease, systematic review, meta-analysis

## Abstract

**Purpose:**

To compare the burden of age-related eye diseases among adults exposed to higher versus lower levels of ambient air pollutants.

**Methods:**

MEDLINE, EMBASE, and Scopus were searched for relevant articles until September 30, 2021. Inclusion criteria included studies of adults, aged 40+ years, that provided measures of association between the air pollutants (nitrogen dioxide, carbon monoxide [CO], sulfur dioxide, ozone [O_3_], particulate matter [PM] less than 2.5 µm in diameter [PM_2.5_], and PM less than 10 µm in diameter [PM_10_]) and the age-related eye disease outcomes of glaucoma, age-related macular degeneration (AMD), or cataract. Pooled odds ratio (OR) estimates and 95% confidence intervals (CIs) were calculated using a random-effects meta-analysis model. PROSPERO registration ID: CRD42021250078.

**Results:**

A total of eight studies were included in the review. Consistent evidence for an association was found between PM_2.5_ and glaucoma, with four of four studies reporting a positive association. The pooled OR for each 10-µg/m^3^ increase of PM_2.5_ on glaucoma was 1.18 (95% CI, 0.95–1.47). Consistent evidence was also found for O_3_ and cataract, with three of three studies reporting an inverse association. Two of two studies reported a null association between PM_2.5_ and cataract, while one of one studies reported a positive association between PM_10_ and cataract. One of one studies reported a positive relationship between CO and AMD. Other relationships were less consistent between studies.

**Conclusions:**

Current evidence suggests there may be an association between some air pollutants and cataract, AMD, and glaucoma.

Ambient air pollution has been recognized as a major contributor to global disease burden and is known to be associated with adverse effects to the pulmonary, cardiovascular, and central nervous systems, as well as age-related eye disease.^1^^–^^5^ Air pollution has been recognized as the most significant environmental threat to human health by the 2018 Environmental Performance Index.^6^ Further, the World Health Organization (WHO) has estimated that 91% of the world's population resides in regions exceeding recommended exposure levels.^7^ As the fifth leading cause of mortality, air pollution is responsible for 4.2 million deaths and 103.1 million disability-adjusted life years lost each year.^1^ Principal sources of air pollution include coal combustion, automotive vehicle emissions, and biofuels used for indoor cooking.^8^ Ambient air pollution is composed of many compounds, including nitrogen dioxide (NO_2_), carbon monoxide (CO), sulfur dioxide (SO_2_), ozone (O_3_), particulate matter (PM) less than 2.5 µm in diameter (PM_2.5_), PM less than 10 µm in diameter (PM_10_), and others.^9^

The eye is directly exposed to air pollution. Research from the developing world has been done that reports that household air pollutants are associated with eye diseases like cataract.^10^^–^^12^ Also, smoking cigarettes is associated with cataract^13^ and age-related macular degeneration (AMD).^14^ However, the risks of ambient air pollutants on age-related eye disease have not been widely studied, and some of the methodology and results have been inconsistent.^10^^–^^14^

The purpose of this study was therefore to synthesize the existing evidence on the global associations of ambient air pollutants (CO, NO_2_, SO_2_, O_3_, PM_2.5_, PM_10_) and age-related eye disease (AMD, cataract, and glaucoma).

## Methods

This systematic review and meta-analysis was conducted in accordance with the process and methods recommended by the Preferred Reporting Items for Systematic Reviews and Meta-Analyses 2020 guidelines.^15^

### Registration and Protocol

Prospero registration ID: CRD42021250078. Deviations from the original study protocol are listed in Supplementary Appendix 1.

### Eligibility Criteria

Eligible studies were those of middle- and older-aged adults, aged 40+ years, that studied the association between air pollutants such as CO, NO_2_, SO_2_, O_3_, PM_2.5_, or PM_10_ and the age-related eye disease outcomes of glaucoma, AMD, or cataract using quantitative effect estimates, such as the risk ratio, odds ratio (OR), hazard ratio (HR), or linear regression coefficient (β) and the respective 95% confidence interval (CI). Only English peer-reviewed studies published prior to September 2021 that used cross-sectional, prospective or retrospective cohort, and case-control study designs were considered eligible for the systematic review. Studies examining the same air pollutant and eye disease that used the same type of regression procedure were considered eligible for inclusion in the meta-analysis.

### Information Sources

MEDLINE, EMBASE, and Scopus were searched for articles that compared higher versus lower levels of environmental air pollutants (CO, NO_2_, SO_2_, O_3_, PM_2.5_, PM_10_) with the outcomes of age-related eye disease (glaucoma, AMD, and cataract) in middle- and older-aged adult populations using the keywords “glaucoma,” “cataract,” “macular degeneration,” “air pollution,” “particulate matter,” “carbon monoxide,” “sulfur dioxide,” “nitrogen dioxide,” and “ozone,” with the appropriate MESH terms until September 30, 2021. A search of the reference lists of included studies and other relevant reviews was also conducted in an attempt to retrieve additional relevant articles.

### Search Strategy

Copies of the peer-reviewed search strategies for MEDLINE, EMBASE, and Scopus are presented in Supplementary Appendix 2.

### Selection Process

The two reviewers (AG/GL) independently screened the titles and abstracts of the identified studies for inclusion and graded them as eligible, ineligible, or potentially eligible based on the prespecified inclusion criteria. The full text of studies deemed potentially eligible based on the title and abstract were then independently reviewed and graded as eligible or ineligible. Conflicts that arose during title and abstract screening were resolved through a full-text review and discussed by the two independent reviewers until a final decision was agreed upon. Full texts from the articles deemed eligible in the title and abstract screening were independently reviewed by the two researchers and graded as eligible or ineligible; any conflicts that arose were resolved through a discussion between the two reviewers until an agreed final decision was reached. A study was included in the systematic review only when both reviewers independently assessed it as satisfying the inclusion criteria based on the full-text review.

### Data Collection Process

Using a standardized data extraction form, the two reviewers (AG/GL) independently extracted data from the included articles. The two reviewers met to discuss any identified discrepancies in the extracted data; disagreements between reviewers were discussed until a consensus had been reached. AG may be contacted to request either raw data or additional data to those reported.

### Data Items

Data abstracted included the following summary data: sample characteristics (including sample size, age of participants, study locations), study design, publication details, air pollutant(s) reported including how they were measured, health outcome(s) reported (AMD, glaucoma, cataract) including how they were measured, the reported associations of the air pollutants with age-related eye disease, and adjustment variables.

### Study Risk of Bias Assessment

At the study level, risk of bias and applicability was independently assessed by the two reviewers (AG/GL) using the Newcastle–Ottawa Scale for assessing the quality of nonrandomized studies in meta-analyses.^16^ Case-control and cohort studies that scored ≥7, 4 to 6, and <4 were considered as low, intermediate, and high risk, respectively, whereas cross-sectional studies that scored ≥7, 6, and ≤5 were considered as low, intermediate, and high risk, respectively. These thresholds were adapted to coincide with previous research assessing risk of bias of nonrandomized studies.^17^^,^^18^ Cohen's κ statistic was used to assess agreement between reviewers.^19^

### Effect Measures

The principal outcome of interest is the association between air pollutants and the health outcomes of glaucoma, AMD, or cataract. Quantitative effect estimates, including both ORs and HRs and the respective 95% CIs, were assessed.

### Synthesis Methods

Data were converted to a tabulated form in order to allow for analysis of results. Studies were grouped by the age-related eye disease reported. For studies grouped by the measured air pollutant and the age-related eye disease that used the same type of regression, effect estimates were converted to the same unit increase (i.e., OR for each 10-µg/m^3^ increase in air pollutant exposure). Pooled OR estimates and 95% CIs were calculated using a random-effects meta-analysis model in which study weights were inversely related to the total variance, and between-study variability was estimated using restricted maximum likelihood. To account for heterogeneity, we performed sensitivity analyses in which the meta-analysis was stratified by study design and by glaucoma assessment method (self-report versus administrative records or ophthalmologic evaluation). In the meta-analyses, the percentage of variance due to heterogeneity was estimated by the *I*^2^ statistic. Analyses were conducted using Stata SE Version 16 (StataCorp, College Station, TX, USA).

### Reporting Bias Assessment

Due to the small number of studies eligible for inclusion in the meta-analysis, it was not feasible to apply statistical tests to assess the potential role of publication bias.

### Certainty Assessment

Studies were independently assessed by the two reviewers (AG/GL) using the Grading of Recommendations, Assessment, Development and Evaluations (GRADE) approach to determine the magnitude of effect and quality of evidence.^20^ All ratings started at a low level of certainty given guidelines for systematic reviews, including only observational studies. Evidence ratings were downgraded based on risk of bias, inconsistency, indirectness, imprecision, and publication bias or upgraded due to large effects, dose–response relationships, or a lack of plausible confounding. GRADE evidence ratings were categorized as very low, low, moderate, or high.

## Results

### Study Selection

**Figure 1.**
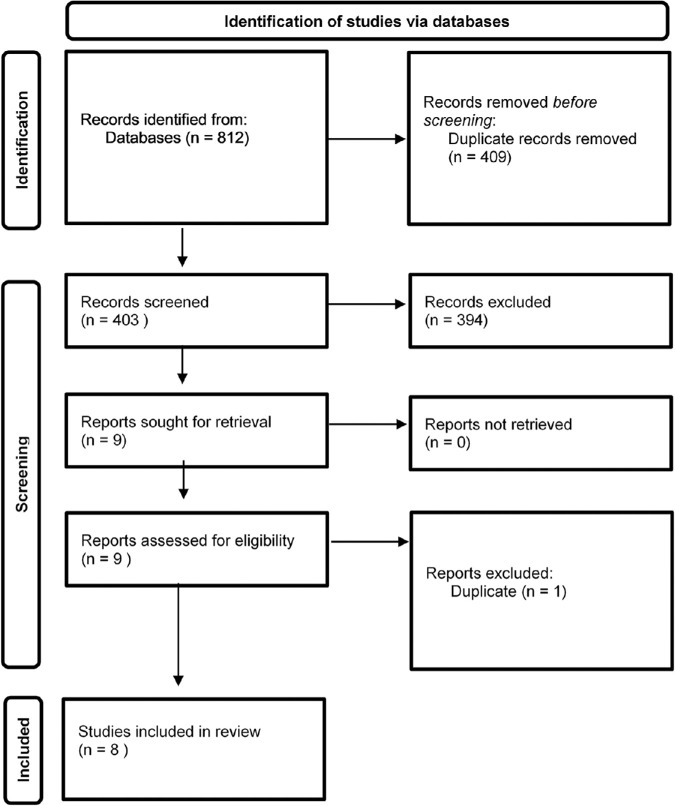
Preferred Reporting Items for Systematic Reviews and Meta-Analyses study flow diagram.

A study flow diagram, which details search and inclusion criteria, is presented in Figure 1. The initial search identified 403 unique articles, which were screened by title and abstract, resulting in 9 articles for full-text review. After inclusion criteria were applied and consensus was reached, one more duplicate article was removed and eight articles (total *N* = 467,566) were included in the review.^3^^–^^5^^,^^21^^–^^25^

**Figure 2.**
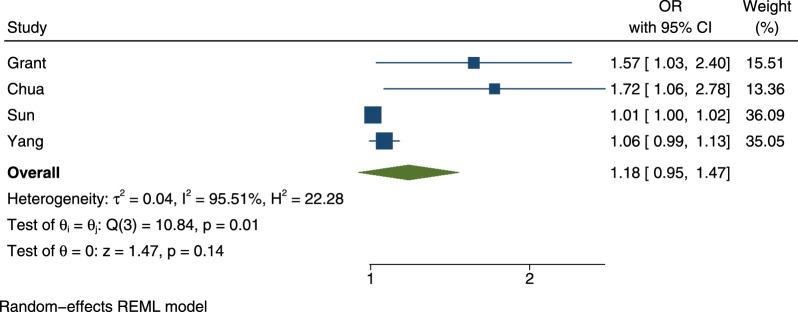
Forest plot of studies included in the meta-analysis.

### Study Characteristics

The characteristics of the included studies are presented in Table 1. Two^4^^,^^5^ of the eight studies were longitudinal. The reported associations of ambient air pollutants with age related eye-disease are summarized in Table 2.

**Table 1.** Characteristics of Studies Included in the Systematic Review

Study	Sample Size	Source of Participants	Age of Participants, y	Investigative Site Location	Study Design	Method of Assessing Exposure to Air Pollution	Method of Measuring Eye Disease Status
Chang et al. (2019)^5^	39,819	Taiwan National Health Insurance Program	50+	Taiwan	Longitudinal population-based study	Eligible patients were those who sought care for an acute respiratory infection. These data were linked with the air pollution levels at the given hospital according to the Taiwan Air Quality Monitoring Database.	ICD-9-CM classification in the Longitudinal Health Insurance Database
Choi et al. (2018)^21^	18,622	Korea National Health and Nutrition Examination Survey	40+	Korea	Cross-sectional population-based study	Air pollution data for the 2 years prior to the ocular exams were collected from national monitoring stations.	Evaluated by ophthalmologists
Chua et al. (2019)^3^	111,370	UK Biobank	40–69	United Kingdom	Cross-sectional population-based study	Air pollution data were obtained from the Small Area Health Statistics Unit. PM_2.5_ exposure was calculated with the land use regression models created by the European Study of Cohorts for Air Pollution Effects project.	Self-reported
Chua et al. (2022)^22^	115, 954	UK Biobank	40–69	United Kingdom	Cross-sectional population-based study	Same as Chua et al. (2019) above	Self-reported
Shin et al. (2020)^4^	115,728	Korean National Insurance Service–National Sample Cohort	50+	Korea	Longitudinal population-based study	Korean Air Pollutants Emission Service in 2002–2015 measured levels every hour.	Diagnosed cataract by ICD-10 criteria (H25, H26) and received cataract surgery (S5119) between 2004 and 2015. Patients with a diagnosed cataract between 2002 and 2003 were excluded.
Sun et al. (2021)^23^	3225	Longitudinal Health Insurance Database 2010 of Taiwan for the 2008–2013 period	65+	Taiwan	Nested case-control study	Taiwan Air Quality Monitoring Database. PM_2.5_ exposure grouped using WHO levels: normal level (<25 µg/m^3^ × exposure months); WHO 1.0 level (≥1 to <1.5 × [25 µg/m^3^ × exposure months]); WHO 1.5 level (1.5 to <2 × [25 µg/m^3^ × exposure months]); and WHO 2.0 level (≥2 × 25 µg/m^3^ × exposure months).	ICD-9-CM classification in the Longitudinal Health Insurance Database
Grant et al. (2021)^24^	29,147	Canadian Longitudinal Study on Aging	45 – 85	Canada	Cross-sectional population-based study	Annual mean PM_2.5_, ozone, sulfur dioxide, and nitrogen dioxide levels for each participant's postal code were estimated using satellite data from the Canadian Urban Environmental Health Research Consortium (CANUE).	Self-reported
Yang et al. (2021)^25^	33,701	The Rural Epidemiology for Glaucoma in China Study	40+	China	Cross-sectional population-based study	A satellite-based model was used to estimate PM_2.5_ concentrations at 1-km resolution, which were assigned to each participant by geocoded home addresses.	Evaluated by ophthalmologists

ICD-9-CM, International Classification of Diseases, 9th Revision, Clinical Modification; ICD-10, International Classification of Diseases, 10th Revision.

**Table 2.** Overview of the Reported Associations of Ambient Air Pollutants With Age-Related Eye Disease From Studies Included in the Systematic Review

Author, Year	Eye Disease(s) Measured	Air Pollutant(s) Measured	Statistical Model	Reported Effect Size	Covariate Adjustment
Chang et al. (2019)^5^	AMD	NO_2_ and CO	Multiple Cox proportional hazards regression	Single-pollutant models: Adjusted HR: 1.91 (95% CI, 1.64–2.23). Exposure: Highest NO_2_ quartile to lowest NO_2_. Adjusted HR: 1.84 (95% CI, 1.57–2.15). Highest CO quartile to lowest quartile exposure.	Age, sex, insurance fee, urbanization, alcoholism, ischemic heart disease, chronic obstructive pulmonary disease, diabetes mellitus, hyperlipidemia, and hypertension
Chua et al. (2022)^22^	AMD	PM_2.5_, PM_10_, NO_2_	Multiple logistic regression analyses	Single-pollutant models: Adjusted OR: 1.08 (95% CI, 1.01–1.16) per IQR (1.07 µg/m^3^) increase in PM_2.5_. Adjusted OR: 0.94 (95% CI, 0.86–1.02) per IQR (2.67 µg/m^3^) increase in PM_10_. Adjusted OR: 0.99 (95% CI, 0.91–1.08) per IQR (12.08 µg/m^3^) increase in NO_2_.	Age, sex, race, Townsend deprivation index, body mass index, smoking status, spherical equivalent refraction
Choi et al. (2018)^21^	Cataract	O_3_, NO_2_, SO_2_, PM_10_	Multiple logistic regression analyses	**All cataract:** Single-pollutant models: Adjusted OR: 0.87 (95% CI, 0.78–0.96) per 0.003-ppm increase in O_3_. Adjusted OR: 0.98 (95% CI, 0.93–1.02) per 0.003-ppm increase in NO_2_. Adjusted OR: 0.81 (95% CI, 0.59–1.10) per 0.003-ppm increase in SO_2_. Adjusted OR: 0.94 (95% CI, 0.85–1.03) per 5-µg/m^3^ increase of PM_10_. Multipollutant models: Adjusted OR: 0.80 (95% CI, 0.69–0.93) per 0.003-ppm increase in O_3_. Adjusted OR: 0.93 (95% CI, 0.85–1.02) per 0.003-ppm increase in NO_2_. Adjusted OR: 0.90 (95% CI, 0.62–1.30) per 0.003-ppm increase in SO_2_. Adjusted OR: 0.91 (95% CI, 0.78, 1.07) per 5-µg/m^3^ increase of PM_10_.	Age, sex, region of residence, education level, income level, smoking, alcohol drinking, hypertension, diabetes mellitus, hypercholesterolemia, myopia, obesity
Shin et al. (2020)^4^	Cataract	PM_2.5_, PM_10_, NO_2_, CO, SO_2_, O_3_	Multiple Cox proportional hazards regression	Single-pollutant models: Adjusted HR: 0.91 (95% CI, 0.77–1.06) PM_2.5_ highest quartile vs. lowest. Adjusted HR: 1.07 (95% CI, 1.03–1.12) PM_10_ highest quartile vs. lowest. Adjusted HR: 1.08 (95% CI, 1.03–1.13) highest NO_2_ quartile vs. lowest. Adjusted HR: 1.03 (95% CI, 0.98–1.07) highest SO_2_ quartile vs. lowest. Adjusted HR: 0.93 (95% CI, 0.89–0.98) highest O_3_ quartile vs. lowest. Adjusted: 0.99 (95% CI, 0.95–1.04) highest CO quartile vs. lowest.	Age, sex, smoking status, income levels, urbanization, comorbidity
Chua et al. (2019)^3^	Glaucoma	PM_2.5_	Multiple logistic regression analyses	Single-pollutant model: Adjusted OR: 1.06 (95% CI, 1.01–1.12) per IQR (1.12 µg/m^3^) increase of PM_2.5_.	Age, sex, race, Townsend deprivation index, BMI, smoking status, spherical equivalent refraction
Sun et al. (2021)^23^	Glaucoma	PM_2.5_	Multiple logistic regression analyses	Single-pollutant model: Adjusted OR: 1.19 (95% CI, 1.05–1.36), per WHO exposure risk. Adjusted OR: 1.67 (95% CI, 1.05–2.66), for WHO 2.0 level.	Sex, age, low income, urbanization level, and comorbidity
Grant et al. (2021)^24^	AMD, cataract, glaucoma	PM_2.5_, O_3_, SO_2_, NO_2_	Multiple logistic regression analyses	Single pollutant models: Adjusted OR glaucoma: 1.14 (95% CI, 1.01–1.29) per IQR (2.9 µg/m^3^) increase of PM_2.5_. Adjusted OR AMD (no visual impairment): 1.00 (95% CI, 0.86–1.15) per IQR increase of PM_2.5_. Adjusted OR AMD (with visual impairment): 1.51 (95% CI, 1.10–2.08) per IQR increase of PM_2.5_. Adjusted OR cataract: 1.06 (95% CI, 0.99–1.14) per IQR increase of PM_2.5_. Multipollutant models: Adjusted OR glaucoma: 1.24 (95% CI, 1.05–1.46) per IQR increase of PM_2.5_. Adjusted OR AMD (no visual impairment): 0.99 (95% CI, 0.82–1.20) per IQR increase of PM_2.5_. Adjusted OR AMD (with visual impairment): 1.41 (95% CI, 0.96–2.08) per IQR increase of PM_2.5_. Adjusted OR cataract: 0.98 (95% CI, 0.90–1.07) per IQR increase of PM_2.5_. Adjusted OR cataract: 0.92 (95% CI, 0.85–0.99) per IQR increase of O_3_.	Age, sex, ethnicity, education, household income, smoking, diabetes, hypertension, province, O_3_, SO_2_, NO_2_
Yang et al. (2021)^25^	Glaucoma	PM_2.5_	Multiple logistic regression analyses	Single-pollutant model: Adjusted OR glaucoma: 1.07 (95% CI, 1.00–1.15) per 10 µg/m^3^ PM_2.5_.	Sex, age, region, disposable income per capita, smoking, hypertension, IOP and lowering IOP treatment

### Risk of Bias in Studies

Risk of bias assessments are presented in Supplementary Table S1. Three studies^4^^,^^21^^,^^25^ were considered to have a low risk of bias (total score ≥7) based on the Newcastle–Ottawa scale adapted for nonrandomized studies. Five studies^3^^,^^5^^,^^22^^–^^24^ were considered to have a medium risk of bias (total score <7) due to low response rates,^3^^,^^22^^,^^24^ low representativeness,^5^ imprecise ascertainment of exposure,^5^ and a lack of adjustment in analyses for lifestyle factors such as smoking status.^23^ Cohen's κ was 0.75, indicating good interrater agreement.

### Results of Individual Studies

Three studies reported on the outcome of AMD.^5^^,^^22^^,^^24^ First, single-pollutant model findings from Chang et al.^5^ suggest that there is an increased risk of AMD among those exposed to higher levels of both CO (HR = 1.84 for the highest quartile; 95% CI, 1.57–2.15) and NO_2_ (HR = 1.91 for the highest quartile; 95% CI, 1.64–2.23). There was no increased risk for the second or third quartiles, indicating a threshold rather than a dose–response effect. Chang et al.^5^ did not present a multipollutant model. Second, single-pollutant model findings from Chua et al.^22^ reported an increased odds of AMD among those exposed to higher levels of PM_2.5_ (OR = 1.08; 95% CI, 1.01–1.16, per interquartile range [IQR] increase in PM_2.5_). However, they did not find any significant association between exposure to PM_10_ or NO_2_ with AMD. Chua et al.^22^ did not present a multipollutant model. Third, Grant et al.^24^ reported that those who lived in areas with higher PM_2.5_ levels were more likely to have visually impairing AMD (OR = 1.51 per IQR; 95% CI, 1.10–2.08) in a single–pollutant model. However, in a multipollutant model, they found a borderline association between exposure to PM_2.5_ and visually impairing AMD (OR = 1.41 per IQR; 95% CI, 0.96–2.08). NO_2_, SO_2_, and O_3_ were not related to AMD in the multipollutant model.

Three studies reported on the outcome of cataracts.^4^^,^^21^^,^^24^ First, results from Choi et al.^21^ found that there was no statistically significant association between PM_10_, NO_2_, or SO_2_ exposure and any cataract in single- or multipollutant models. However, the results for NO_2_ differed by cataract subtype. NO_2_ (per 0.003-ppm increase) showed a protective association with anterior subcapsular cataracts (OR = 0.69; 95% CI, 0.52–0.93) in the multipollutant model only and a protective association with nuclear cataracts in the single-pollutant (OR = 0.89; 95% CI, 0.83–0.94) and multipollutant models (OR = 0.88; 95% CI, 0.79–0.97). Choi et al.^21^ also found a statistically significant protective relationship between O_3_ exposure (per 0.003-ppm increase) and any cataract that was consistent across single-pollutant (OR = 0.87; 95% CI, 0.78–0.96) and multipollutant (OR = 0.80; 95% CI, 0.69–0.93) models. When assessing by cataract subtype, there was a statistically significant relationship between O_3_ exposure with nuclear cataract in both single-pollutant (OR = 0.89; 95% CI, 0.83–0.94) and multipollutant (OR = 0.73; 95% CI, 0.61–0.86) models but not for anterior subcapsular, posterior subcapsular, or cortical cataracts.^21^ Second, in contrast to Choi et al.,^21^ Shin et al.^4^ found that higher exposure to NO_2_ (HR = 1.08 for the highest quartile; 95% CI, 1.03–1.13) was associated with an increased risk of cataract. They also found that PM_10_ was associated with an increased risk of cataract (HR = 1.07 for the highest quartile; 95% CI, 1.03–1.12). Also in contrast to Choi et al.,^21^ those in the second and third quartiles of SO_2_ exposure had a higher cataract incidence, with HRs of 1.07 (95% CI, 1.02–1.11) for the second quartile and 1.05 (95% CI, 1.00–1.09) for the third quartile. The fourth quartile did not have a higher incidence (HR = 1.03; 95% CI, 0.98–1.07). Shin et al.^4^ found a protective association between O_3_ and cataract (*P* value for linear trend = 0.013). There was no increased risk of cataract reported for those with greater exposure to PM_2.5_. Finally, Grant et al.^24^ reported a borderline association between PM_2.5_ and cataract (OR = 1.06 per IQR; 95% CI, 0.99–1.14) in a single-pollutant model. However, they did not find an increased odds of cataract among those with greater exposure levels to PM_2.5_ (OR = 0.98 per IQR; 95% CI, 0.90–1.07) in the multipollutant model. Further, higher O_3_ levels were reported to be inversely associated with cataract in a multipollutant model (OR = 0.92 per IQR; 95% CI, 0.85–0.99).^24^ No association was found for NO_2_ or SO_2_ with cataract in a multipollutant model.

Finally, four studies reported on the outcome of glaucoma.^3^^,^^23^^–^^25^ First, Chua et al.^3^ found that PM_2.5_ exposure was associated with a higher odds of glaucoma in a single-pollutant model (OR = 1.06; 95% CI, 1.01–1.12, per IQR increase of PM_2.5_). Second, Sun et al.^23^ found that exposure to PM_2.5_ was associated with primary open-angle glaucoma in a single-pollutant model. These findings were statistically significant per increase in WHO exposure level of PM_2.5_ (OR = 1.19; 95% CI, 1.05,1.36) and comparing the highest exposure category to the reference level (OR = 1.67; 95% CI–1.05–2.66). Grant et al.^24^ also found that increased PM_2.5_ level (per IQR) was significantly associated with glaucoma in both the single-pollutant (OR = 1.14; 95% CI 1.01–1.29) and multipollutant (OR = 1.21; 95% CI 1.04–1.42) models. NO_2_, SO_2_, and O_3_ were not associated with glaucoma in a multipollutant model. Finally, Yang et al.^25^ found that each 10-µg/m^3^ increment in PM_2.5_ exposure was associated with a higher odds of glaucoma in a single-pollutant model (OR = 1.07; 95% CI, 1.00–1.15).

### Results of Syntheses

Four studies examined the association of glaucoma and PM_2.5_ exposure using the same type of regression and were therefore eligible for inclusion in the meta-analysis. The forest plot of the studies included in the meta-analysis is presented in Figure 2. The pooled OR for each 10-µg/m^3^ increase of PM_2.5_ on glaucoma was 1.18 (95% CI, 0.95–1.47). The *I*^2^ value was 96%, indicating high heterogeneity. In our meta-analysis stratified by study design, the pooled OR for each 10-µg/m^3^ increase of PM_2.5_ on glaucoma in cross-sectional studies was 1.33 (95% CI, 0.95–1.84) and the *I*^2^ value reduced to 67% (Supplementary Fig. S1). As only one study utilized a case-control design, we are unable to report a pooled OR for this design. In our meta-analysis stratified by the glaucoma assessment method, the pooled OR for each 10-µg/m^3^ increase of PM_2.5_ was 1.63 (95% CI, 1.19–2.24) for self-reported glaucoma and 1.02 (95% CI, 0.98–1.07) for glaucoma determined by an ophthalmologic exam or health administrative records. In both models, the *I*^2^ value reduced substantially (Supplementary Figs. S2 and S3).

### Certainty of Evidence

Certainty of evidence assessments are presented in Table 3. Overall, we are moderately confident in the effect estimates of two^4^^,^^21^ of the eight included studies. These studies were upgraded to medium for having a dose–response relationship and were not downgraded for bias. The other six^3^^,^^5^^,^^22^^–^^25^ studies had a low certainty of evidence. Due to the imprecise exposure measures of air pollution at regional rather than individual levels, there is a possibility of measurement error in all studies; however, we believe this would cause a dilution of the true effects.

**Table 3.** GRADE Assessments of Certainty

Study	GRADE Assessment of Certainty	Reasons for Downgrade or Upgrade
Chang et al. (2019)^5^	Low	Risk of bias (−1) Dose response (+1)
Chua et al. (2022)^22^	Low	Risk of bias (−1) Dose response (+1)
Choi et al. (2018)^21^	Moderate	Dose response (+1)
Shin et al. (2020)^4^	Moderate	Dose response (+1)
Chua et al. (2019)^3^	Low	Risk of bias (−1) Dose response (+1)
Sun et al. (2021)^23^	Low	Risk of bias (−1) Dose response (+1)
Grant et al. (2021)^24^	Low	Risk of bias (−1) Dose response (+1)
Yang et al. (2021)^25^	Low	Risk of bias (−1) Dose response (+1)

## Discussion

Overall, findings from the systematic review suggest a potentially increased risk of AMD among individuals exposed to higher levels of CO^5^ and PM_2.5_.^22^^,^^24^ While only one study reported on CO and AMD,^5^ the association is biologically plausible as increased CO exposure may induce an accumulation of oxidative stress in the retina,^26^ which is particularly susceptible to this cellular damage due to its exposure to visible light, high proportion of polyunsaturated fatty acids, and high oxygen consumption,^27^ thereby furthering the progression or development of AMD. Both Chua et al.^22^ and Grant et al.^24^ reported that higher exposures of PM_2.5_ were associated with AMD or associated in a borderline fashion with visually impairing AMD, respectively. It is possible that PM_2.5_ affects predominantly the late AMD neovascularization process, which affects visual acuity. Previous research findings support this idea as exposure to PM_2.5_ has been reported to be associated with impaired endothelial function and proangiogenic molecules, which are known biomarkers of oxidative stress.^28^ The evidence was inconsistent for NO_2_, with one study reporting an association^5^ and two not reporting one.^22^^,^^24^ Only one study examined the relationship between PM_10_ and AMD with null results.^22^ Since limited evidence is available for each air pollution exposure, further studies are needed to ascertain the relationship between air pollution and AMD.

There is inconsistent evidence regarding air pollution exposure and cataracts. For PM_10_ and SO_2,_ the results were inconclusive, as Choi et al.^21^ found no difference in the odds of cataract with higher exposure levels to these pollutants, while Shin et al.^4^ reported increased risks of cataract that were nonlinear by quartile of exposure.^4^^,^^21^ Results were also contradictory for NO_2_ and cataract, as Choi et al.^21^ suggested a protective association with some subtypes of cataract, Shin et al.^4^ found an increased risk of cataract in a dose-dependent manner, and Grant et al.^24^ found no association. More consistent evidence was found for ozone and cataract. Three studies have reported a protective association between higher levels of ozone and cataract.^4^^,^^21^^,^^24^ A potential mechanism suggested by Choi et al.^21^ to explain the reported protective association between O_3_ and cataract may be related to the ability of O_3_ to block ultraviolet light and thereby reduce oxidative damage. To date, only two studies have reported on the association of PM_2.5_ exposure and cataract. Shin et al.^4^ found no statistically significant association, while Grant et al.^24^ found a borderline association in a single-pollutant model and no association in a multipollutant model. Prior research has indicated that cataract risk factors differ by cataract subtype.^29^ Given some of the inconsistency in the findings for cataract, further studies should differentiate between different types of cataract. Choi et al.^21^ attempted to look at cataract subtypes, but their sample size was quite limited for some rarer subtypes like anterior and posterior subcapsular. If the risk is different by cataract subtype, combining all subtypes together may obscure the true effect.

Finally, the four studies that examined glaucoma in this review all reported higher burdens of glaucoma among those with higher exposure levels to PM_2.5_.^3^^,^^23^^–^^25^ Emerging evidence from an in vitro study conducted on human trabecular meshwork cells^30^ provides evidence for the biologic plausibility of these associations. Li et al.^30^ found that exposure to PM_2.5_ resulted in increased oxidative stress in the intraocular tissues and in the subsequent activation of NACHT, LRR, and PYD domains-containing protein 3 (NLRP_3_) inflammasome-mediated pyroptosis in trabecular meshwork cells. Only Grant et al.^24^ reported on the association between other pollutants and glaucoma. Null associations were found between NO_2_, SO_2_, and O_3_ and glaucoma in the multipollutant model. Adjusting for these other pollutants strengthened the relationship between PM_2.5_ and glaucoma. When study results were pooled in the meta-analysis, however, the association between exposure to PM_2.5_ and glaucoma no longer reached statistical significance, and significant heterogeneity was measured. As these studies used different methodologies, analysis methods, and adjustment of confounding variables, the accuracy of the estimate derived in the meta-analysis is unclear. Stratifying the meta-analysis by study design resulted in an increased OR estimate of the association between PM_2.5_ and glaucoma to borderline significance for cross-sectional studies, but moderate heterogeneity was still measured. Stratifying the meta-analysis by glaucoma assessment method resulted in an increased OR estimate of the association between PM_2.5_ and International Classification of Diseases (ICD)-coded or ophthalmologist-evaluated glaucoma to borderline significance, as well as in a statistically significant OR estimate of the association between PM_2.5_ and self-reported glaucoma and reduced heterogeneity measures to 0%. Therefore, the difference in glaucoma assessment methods appears to be the factor contributing most significantly to heterogeneity in this study.

Strengths of the current study include the use of recent evidence, in which large sample sizes were utilized. Due to the scarcity of the data on this topic and the significant heterogeneity among the included studies, we were unable to pool most study results into a meta-analysis. Limitations were also significant among the included studies. The major limitation impacting the robustness of our study findings is that six^3^^,^^5^^,^^22^^–^^25^ of the eight included studies had low certainty of evidence ratings. In accordance with the GRADE guidelines for systematic reviews including only observational studies, all eight studies started with a low level of certainty ratings. The evidence ratings of all studies were first upgraded to moderate for reporting dose–response relationships. Two^4^^,^^21^ of these studies remained at moderate certainty of evidence ratings due to low risks of bias. The other six^3^^,^^5^^,^^22^^–^^25^ studies were again downgraded back to low due to risks of bias resulting from low response rates,^3^^,^^22^^,^^24^ nonrandom sample,^5^ imprecise ascertainment of exposure,^5^ imprecise ascertainment of outcome,^3^^,^^22^^,^^24^ limited data on lifestyle confounders,^25^ or a lack of adjustment in analyses for important confounding factors.^23^ Five studies^3^^,^^21^^,^^22^^,^^24^^,^^25^ utilized a cross-sectional design, and therefore it is not possible to delineate the temporality of exposure to outcome. Further, there was significant heterogeneity in the definition of eye disease status, in which three studies^3^^,^^22^^,^^24^ out of eight used self-reported data for the ascertainment of age-related eye disease, which is known to have limited validity^31^ and may lead to misclassification. For example, as glaucoma is often asymptomatic until late in the disease process, the rate of undiagnosed glaucoma is often high and therefore highly underreported. Further, people treated for ocular hypertension may mistakenly think they have glaucoma because they take pressure-lowering eye drops. Also, the definition of cataract was inconsistent between studies, defined using administrative records,^4^ clinical examination,^21^ or by self-report.^24^ Therefore, inconsistent evidence on associations of air pollutants and cataract may be related to the limited validity of the self-report of cataract and the differing severity of cataract among those people who have had cataract surgery compared to those with cataract who have not yet had surgery. Misclassification of eye disease, however, would likely be nondifferential in that those exposed to differing levels of air pollutants would have similar likelihoods of accurately reporting their eye disease status. Other limitations of the included studies include low response rates^3^^,^^22^^,^^24^ and therefore a greater risk of selection bias. Also, a common limitation of the included studies was that information on air pollution exposure was based on the location of the hospital where patients sought treatment or the patient's location of residence so if a person does not spend much time at their residence or near the hospital where they sought treatment, it is likely some measurement error would occur.^32^^,^^33^ In addition, as only studies conducted in Canada, rural areas of China, Korea, Taiwan, and the United Kingdom have been conducted thus far, the generalizability of the study results to other regions and countries remains unclear. Finally, an issue complicating the interpretation of study results for six^3^^–^^5^^,^^22^^,^^23^^,^^25^ of the included studies was the reliance on only single-pollutant models, which may be affected by confounding by other pollutants. Multipollutant models are less likely to be affected by confounding but they may suffer from other biases.^32^

To conclude, an increased risk of AMD was reported among individuals exposed to higher levels of CO and perhaps PM_2.5_. There appears to be a protective association of O_3_ exposure and cataract. Increased PM_2.5_ exposure was also found to be associated with glaucoma. These associations as well as those of NO_2_, PM_10_, and SO_2_ with age-related eye disease should be confirmed using longitudinal data and potential mechanisms should be explored by investigating interactions with genetic factors or inflammatory markers that may be involved in the causal pathway.^5^

## Supplementary Material

Supplement 1
