# Supplementary material for: Ambient Air Pollution and Age-Related Eye Disease: A Systematic Review and Meta-Analysis
Source: Invest Ophthalmol Vis Sci. 2022 Aug 12;63(9):17. doi: 10.1167/iovs.63.9.17 (PMC9396677; doi:10.1167/iovs.63.9.17)
Supplement: Supplement 1 [file iovs-63-9-17_s001.pdf]

SUPPLEMENTARY MATERIAL

S-Appendix 1: Deviations from study protocol

1. Patient population: After our initial full-text screen, we identified an additional relevant article that used a case-control study design. As such, we broadened the inclusion criteria to include this study type.
2. Search strategy end date: Extended from April until September 2021.
3. Sensitivity analyses: As we were unable to perform meta-regression analyses to account for heterogeneity as indicated in our protocol, we performed a stratified meta-analysis to account for study design.

S-Appendix 2. Peer-reviewed search strategies

Medline

1	Glaucoma/
2	glaucoma*.ti,ab.
3	Cataract/
4	cataract*.ti,ab.
5	Macular Degeneration/
6	(macular adj1 degeneration).ti,ab.
7	1 or 2 or 3 or 4 or 5 or 6
8	Air Pollution/
9	(air adj1 pollution).ti,ab.
10	8 or 9
11	Particulate Matter/
12	(particulate adj1 matter).ti,ab.
13	Carbon Monoxide/
14	(Carbon adj1 monoxide).ti,ab.
15	Ozone/
16	ozone*.ti,ab.
17	Nitrogen Dioxide/
18	(nitrogen adj1 dioxide).ti,ab.
19	Sulfur Dioxide/
20	(sulfur adj1 dioxide).ti,ab.
21	11 or 12 or 13 or 14 or 15 or 16 or 17 or 18 or 19 or 20
22	7 and 10
23	7 and 21
24	22 or 23

Embase

1	glaucoma/
2	glaucoma*.ti,ab.
3	cataract/
4	cataract*.ti,ab.
5	macular degeneration/
6	(macular adj1 degeneration).ti,ab.
7	1 or 2 or 3 or 4 or 5 or 6
8	air pollution/
9	(air adj1 pollution).ti,ab.
10	8 or 9

11	particulate matter/
12	(particulate adj1 matter).ti,ab.
13	carbon monoxide/
14	(Carbon adj1 monoxide).ti,ab.
15	ozone/
16	ozone*.ti,ab.
17	nitrogen dioxide/
18	(nitrogen adj1 dioxide).ti,ab.
19	sulfur dioxide/
20	(sulfur adj1 dioxide).ti,ab.
21	11 or 12 or 13 or 14 or 15 or 16 or 17 or 18 or 19 or 20
22	7 and 10
23	7 and 21
24	22 or 23

Scopus

((TITLE-ABS-KEY (glaucoma)) OR (TITLE-ABS-KEY (cataract)) OR (TITLE-ABS-KEY ("macular degeneration"))) AND ((TITLE-ABS-KEY ("particulate matter")) OR (TITLE-ABS-KEY ("nitrogen dioxide")) OR (TITLE-ABS-KEY ("sulfur dioxide")) OR (TITLE-ABS-KEY ("carbon monoxide")) OR (TITLE-ABS-KEY (ozone)) OR (TITLE-ABS-KEY ("air pollution")))

S- Table 1. Newcastle Ottawa Risk of Bias Assessments

Study	Risk of bias assessment			
	**Selection (0-4)**	**Comparability (0-2)**	**Outcome (0-3)**	**Total Score (Cohort)**
Chang et al. (2019)[5]	2	1	3	6/9
Shin et al. (2020)[4]	4	2	3	9/9
	**Selection (0-4)**	**Comparability (0-2)**	**Outcome (0-3)**	**Total Score (Case-control)**
Sun et al. (2021)[23]	3	1	2	6/9
	**Selection (0-4)**	**Comparability (0-2)**	**Outcome (0-2)**	**Total Score (Cross-Sectional)**
Choi et al. (2018)[21]	4	2	2	8/8
Chua et al. (2019)[3]	3	2	1	6/8
Chua et al. (2021)[22]	3	2	1	6/8
Grant et al. (2021)[24]	3	2	1	6/8
Yang et al. (2021)[25]	4	2	2	8/8

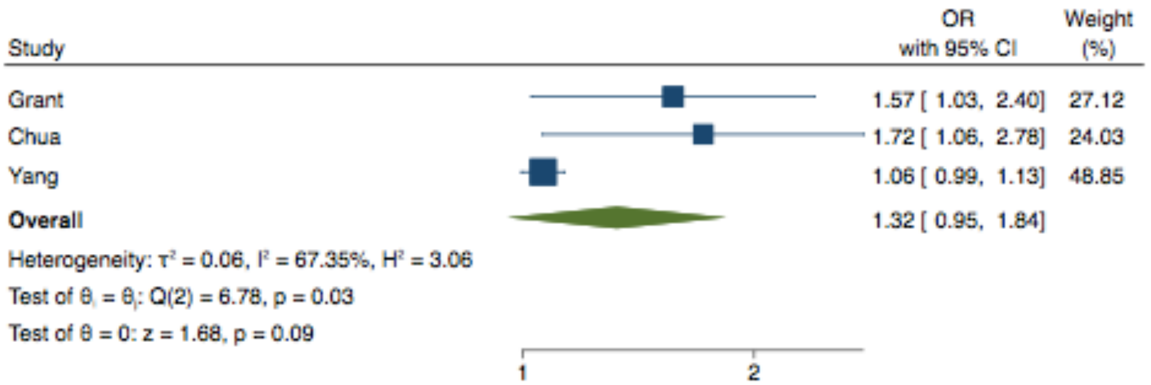

S-Figure 1. Forest plot of studies limited to those with a cross-sectional design

OR= odds ratio; CI= confidence interval

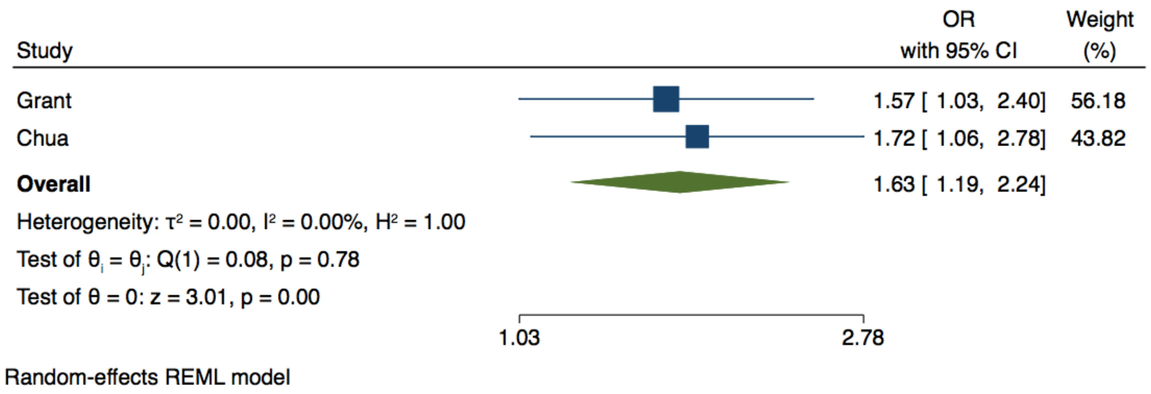

S-Figure 2. Forest plot of studies limited to those using self-reported glaucoma

OR= odds ratio; CI= confidence interval

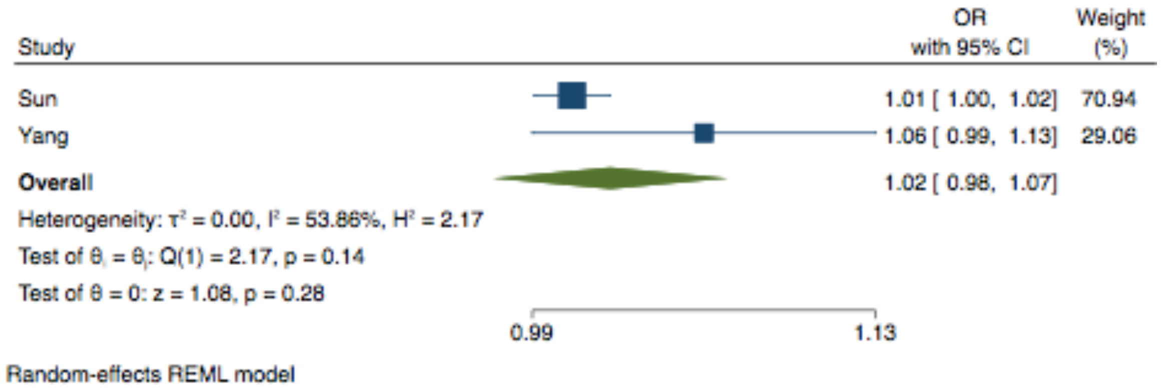

S-Figure 3. Forest plot of studies limited to those having glaucoma determined by health administrative records or by ophthalmological exam using international standards of glaucoma classification

OR= odds ratio; CI= confidence interval
